# MicroRNA‐132 suppresses migration and invasion of renal carcinoma cells

**DOI:** 10.1002/jcla.22969

**Published:** 2019-10-17

**Authors:** Yi Yu, Wenbao Lu, Xinmin Zhou, Hua Huang, Shaochen Shen, Lian Guo

**Affiliations:** ^1^ Department of Urology The Second Affiliated Hospital of Nanchang University Nanchang Jiangxi Province China; ^2^ Department of Urology The Affiliated Hospital of Jiujiang University Clinical Medical College Jiujiang Jiangxi Province China; ^3^ Department of Urology Duchang County Hospital of Traditional Chinese Medicine Duchang Jiangxi Province China; ^4^ Department of Anesthesia The Second Affiliated Hospital of Nanchang University Nanchang Jiangxi Province China

**Keywords:** FOXM1, miRNA‐132, MMP‐2, MMP‐9, renal cell carcinoma

## Abstract

**Background:**

The aim of this study was to explain the effects of microRNA‐132 in renal cell carcinoma by regulating FOXM1 expression.

**Methods:**

Thirty patients with renal cell carcinoma admitted to our hospital were enrolled, and their adjacent normal tissues and cancer tissues were taken. The expression of microRNA‐132 was measured by in situ hybridization (ISH) and RT‐PCR, and the expression of FOXM1 was evaluated by RT‐PCR and immunohistochemistry (IHC), and the correlation between microRNA‐132 and FOXM1 was analyzed. In the cell experiment, the KETR‐3 cells were divided into three groups: Negative control (NC) group were treated with nothing; blank (BL) group were transfected with empty vector; and microRNA‐132 (miRNA) group were transfected with microRNA‐132. The cell invasion and migration abilities among groups were assessed by transwell and wound healing assays. The expression levels of related proteins (FOXM1, MMP‐2, MMP‐9, VEGF‐alpha, and uPAR) were determined by Western blot.

**Results:**

Depending on clinical data, we found that FOXM1 protein expression of renal cell carcinoma tissues was higher than that in adjacent normal tissues. MiRNA‐132 was negative correlation with FOXM1. In vitro, the number of invasive cells and wound healing rate in the microRNA group were significantly suppressed than those in the NC group (*P* < 0.05, respectively). In the Western blot assay, the results showed that the protein expression levels of FOXM1, MMP‐2, MMP‐9, VEGF‐α, and uPAR were significantly inhibited in the miRNA group compared with the NC group (*P* < 0.05, respectively).

**Conclusion:**

miRNA‐132 had anti‐tumor effects in renal cell carcinoma by suppressing FOXM1 expression.

**Abbreviations**
BCIP: 5‐bromo‐4‐chloro‐3‐indolyl phosphate
BL: blank
DAB: 3,3'‐diaminobenzidine
DMEM: Dulbecco's Modified Eagle Medium
FOXM1: Forkhead box M1
GAPDH: Glyceraldehyde‐3‐phosphate dehydrogenase
IHC: immunohistochemistry
ISH: in situ hybridization
miRNA‐132: microRNA‐132
MMP‐2/9: matrix metalloprotein‐2/9
MTT: 3‐(4,5‐Dimethylthiazol‐2‐yl)‐ 2,5‐diphenyltetrazolium bromide
NBT: nitro‐blue‐tetrazolium
NC: negative Control
OD: optical density
PBS: phosphate‐buffered saline
RCC: renal cell carcinoma
RPMI: Roswell Park Memorial Institute
RT‐PCR: reverse transcription polymerase chain reaction
SD: standard deviation
SSC: saline sodium citrate
uPAR: urokinase plasminogen activator receptor
UTR: untranslated region
VEGF2‐α: vascular endothelial growth factor‐α

## 1 INTRODUCTION

Renal cell carcinoma (RCC) is one of the most common malignant tumors of urinary system. The incidence of RCC is increasing year by year.^1^ There are no specific symptoms in early stage of RCC. Most patients with advanced renal cancer have distant metastasis.^2^ Surgery is still the main treatment for renal cancer, because chemotherapy, radiotherapy, and biological targeted therapy are ineffective.^3^ The prognosis of RCC is poor, especially for distant metastasis, and the 5‐year survival rate of RCC is less than 10%.^4^ The cause of RCC is not clear. It is presumed to be related to heredity, hypertension, smoking, and chemical exposure.^5^ There is an urgent need to find molecular markers related to the pathogenesis and early diagnosis of RCC.

MicroRNAs (miRNAs) are about 22‐24 nucleotides in length that encode single‐stranded RNA molecules.^6^ miRNAs bind to the 3' untranslated regions (3'UTR) of mRNA in the target area resulting in the posttranscriptional regulation of gene expression. Therefore, miRNAs play a role in regulating gene expression that is widely involved in cell viability, differentiation and apoptosis, and tumor development.^7^, ^8^ In the course of tumor development, the miRNAs associated with tumorigenesis will change.^9^

Previous studies have indicated that miRNA‐132 is abnormally expressed in some cancers.^10^, ^11^, ^12^, ^13^, ^14^ However, there are no reports on the correlation between miRNA‐132 and RCC. In the present study, we firstly evaluated the expression of miRNA‐132 in adjacent normal and cancer tissues from 30 patients with RCC. And then, we discussed the effects and mechanism of miRNA‐132 in the RCC cell line KETR‐3 cells.

## 2 MATERIAL AND METHODS

### 2.1 Sample and clinical data

The samples were collected from 30 RCC patients, including 16 males and 14 females (aged 45 ± 5.62 years old) who were treated in our hospital from August 2014 to March 2016. Adjacent normal tissues more than 4 cm above the lesion were collected. After removing the specimen, the tissues were divided into two parts: one was quickly protected as RNA and stored in liquid nitrogen within 24 hours. The other part was saved in 4% paraformaldehyde and embedded in paraffin. Then, 4‐µm‐thick sections were dewaxed to distilled water. Consistent with ethical requirements, the written consent was obtained from each participant after providing a clear and thorough explanation of the study. All experiments were done with the approval of Human Health Ethics Committee (No.2014‐07‐12).

### 2.2 In situ hybridization

Samples were dewaxed, hydrated, and washed by phosphate‐buffered saline (PBS) (5 seconds × 2 times). Samples were cultured with 0.1 mol/L HCl for 10 minutes and washed using PBS (5 seconds× 2 times). After drying and dropping the protease K (1:10) at room temperature for 2 minutes, samples were washed by PBS (5 seconds× 2 times), fixed in 4% paraformaldehyde at room temperature for 10 minutes, and washed by PBS (5 seconds × 2 times) at room temperature, followed by 70% solid solution at 80°C for 10 minutes. Then, samples were dehydrated in 90% ethanol for 15 seconds; the suspension solution was covered with a sealing film and then placed in the wet box (42°C, 22 hours). Slices were dipped in the sealing film at 50°C by 5× saline sodium citrate (SSC), washed by 50% formamide‐2× SSC for 30 minutes, 2× SSC for 15 minutes (2 times), 0.1× SSC for 15 minutes, PBS for 5 seconds, and Buffer I for 5 seconds, respectively. Slices were closed by using horse serum (1:100) at room temperature for 30‐60 minutes and incubated with anti‐Dig‐Ag (1:500) at room temperature for 1 hour, then washed by Buffer I (15 minutes × 3 times) and Buffer III (1 minutes × 2 times). Slices were then covered with nitro‐blue‐tetrazolium/5‐bromo‐4‐chloro‐3‐indolyl phosphate (NBT/BCIP) and mounted with glycerol gelatin. The positive cells in each group were quantitatively analyzed by mias‐2000 color image analysis system.

### 2.3 Immunohistochemistry

The sections were treated with conventional xylene and hydrated at various levels of ethanol. A certain amount of pH 6 citrate buffer (Beijing Jinqiao Biological Technology Co., Ltd.) was added in microwave box for antigen repair (3 minutes × 2 times), then cooled to room temperature for 40 minutes. Each slice was added with one drop of 3% H_2_O_2_ and incubated at room temperature for 10 minutes. Slices were covered with antibody (dilution of 1:100) overnight at 4°C in the refrigerator. Then, slices were labeled with horseradish enzyme second antibody (Beijing Jinqiao Biological Technology Co., Ltd.) at 4°C for 30‐40 minutes. Each section was cultured with one drop of freshly prepared 3,3'‐diaminobenzidine (DAB) solution for 20 minutes, stained with light hematoxylin for 30 seconds, rapidly dehydrated by using ethanol (85%, 1 minutes; 95%,1 minutes; 100%, 1 minutes, respectively), and sealed with xylene transparent, neutral resin sheet. Results were observed after drying.

### 2.4 Reverse transcription polymerase chain reaction

The total RNA was extracted by TRIzol, and the integrity and purity of RNA were measured according to the instructions of Hairpin‐it^TM^ qPCR Quantitation Kit. Reverse transcription polymerase chain reaction (RT‐PCR) was performed using PrimeScript reverse transcription kit. SYBR real‐time fluorescent quantitative kit was used for real‐time quantitative PCR detection. The reaction was performed in 25 µL system, and reaction condition was as follows: 50°C for 30 minutes, 94°C for 1 minute, 57°C for 1 minute, and 72°C for 7 minutes. Glyceraldehyde‐3‐phosphate dehydrogenase (GAPDH) and U6 sn‐RNA were considered as references in this study. The primer sequence were as follows: miRNA‐132: F: 5'‐CCAGCATAACAGTCTACAGCCA‐3'; R: 5'⁃TATGGTTGTTCACGACTCCTTCAC‐3'; FOXM1: F: 5' ‐CACCCCAGTGCCAACCGCTACTTG‐3'; R: 5'‐AAAGAGGAGCTATCCCCTCCTCAG‐3'; U6‐snRNA: F: 5'‐ATTGGAACGATACAGAGAAGATT‐3'; R: 5'‐GGAACGCTTCACGAATTTG‐3'; GAPDH: F: 5'‐TCCATGACAACTTTGGCATTGTGG‐3'; R: 5’‐ GTTGCTGTTGAAGTCGCAGGAGAC‐3’. The gene expression value was calculated using the 2^−(ΔΔCt)^ method.^15^

### 2.5 Cell culture and grouping

A whole medium suitable for cell growth, contained 90% Dulbecco's Modified Eagle Medium (DMEM), 1% glutamine, 1% streptomycin, and 10% fetal bovine serum. The RCC cell line KETR‐3 cells at the logarithmic growth phase were placed in culture dish with 4 mL medium and cultured in 37°C, suitable CO_2_ concentration (volume fraction 5%) and PH box. The sixth generation of KETR‐3 cell line was used for the experimental study, and the medium was changed every 2 days. The KETR‐3 cells were divided into three groups: NC group: the KETR‐3 cells were transfected with miRNA‐132 negative control (5'‐AACGCTTCACGAATTTGCGT‐3') at concentration of 50 nmol/L; BL group: the KETR‐3 cells were treated with nothing; miRNA group: the KETR‐3 cells were transfected with miRNA‐132 mimics (5'‐ACCGTGGCTTTCGATTGTTACT‐3') at concentration of 50 nmol/L according to previous study.^16^

### 2.6 MTT assay

The KETR‐3 cells in logarithmic growth phase were inoculated into 96‐well plate (2 × 10^3^/well) for 3‐(4,5‐Dimethylthiazol‐2‐yl)‐2,5‐diphenyltetrazolium bromide (MTT) assay. There were five repeats in every group. The cells in different groups were cultured in the incubator (37°C, 5% CO_2_) for 48 hours. After 20 μL MTT was added into wells and incubated for 1 hour, a value was measured at 490 nm by using the microplate reader. Then, the cell viability in different groups was measured depending on the optical density (OD) values.

### 2.7 Transwell assay

The cells in logarithmic growth phase in each group were cultured in serum‐free medium for 12 hours. The single cell suspension was made by trypsin digestion, and the cell density was adjusted to 1.5 × 10^5^/mL. The melted matrix at 4℃ was diluted and added to the transwell chamber, incubated at 37°C for 30 minutes, and 200 μL cell suspension was added to the chamber to avoid bubbles. 600 μL Roswell Park Memorial Institute (RPMI) 1640 containing 15% fetal bovine serum (FBS) was added to the corresponding hole in 24‐hole plates and terminated after 48 hours of incubation. Cells were washed with PBS at the room temperature for two times, and residual matrix glue was wiped by using swabs. The first chamber was fixed in 4% formaldehyde solution for 15 minutes, stained with 0.1% crystal violet staining for 20 minutes, finally washed to remove the residual dye. After drying, cells were observed by inverted microscope and photographed by using manual microscopic counting method. The cell number of four random fields was used for statistics.

### 2.8 Wound healing assay

At the bottom of 6‐hole plates, there was a gun head covered with cells, and after being scratched, the liquid was changed. Take a picture after 48 hours of scratching and calculate the scratch width in Image Pro Plus 6 (Media Cybernetics). Each group established three holes.

### 2.9 Western blotting assay

The six holes were cleaned with PBS for two times; each hole was added with 150 μL lysis liquid (RIPA: protease inhibitor = 4:1), placed on the ice for 30 minutes, and centrifuged at 11 000 *g*/min for 30 minutes at 4°C The protein content of each sample was determined by bovine serum albumin (BCA) quantitative method. A total of 50 μg sample was added to every lane with conventional gel electrophoresis containing 8%‐12% polyacrylamide. The membrane was closed with BSA and then subjected to primary antibodies and secondary antibodies, followed by immunochemical chemiluminescence. GAPDH was used as an internal control.

### 2.10 Statistical analysis and methods

Three independent experiments were performed for each assay in this study. The data are expressed as mean ± standard deviation ($\bar{X}$ ± SD), and statistical method is selected by t test or chi‐square test. Multiple comparisons were analyzed by using analysis of variance (ANOVA) in SPSS software version 17.0 (SPSS Inc). Enumeration data were compared by chi‐square test. Pearson's method was used in correlation test. The data were processed by GraphPad Prism 6 software (GraphPad Software, Inc), and all the tests were two‐sided; *P* < 0.05 was found to be statistically significant.

## 3 RESULTS

### 3.1 The expression levels of miRNA‐132 and FOXM1 in adjacent normal tissues and RCC tissues

**Figure 1:**
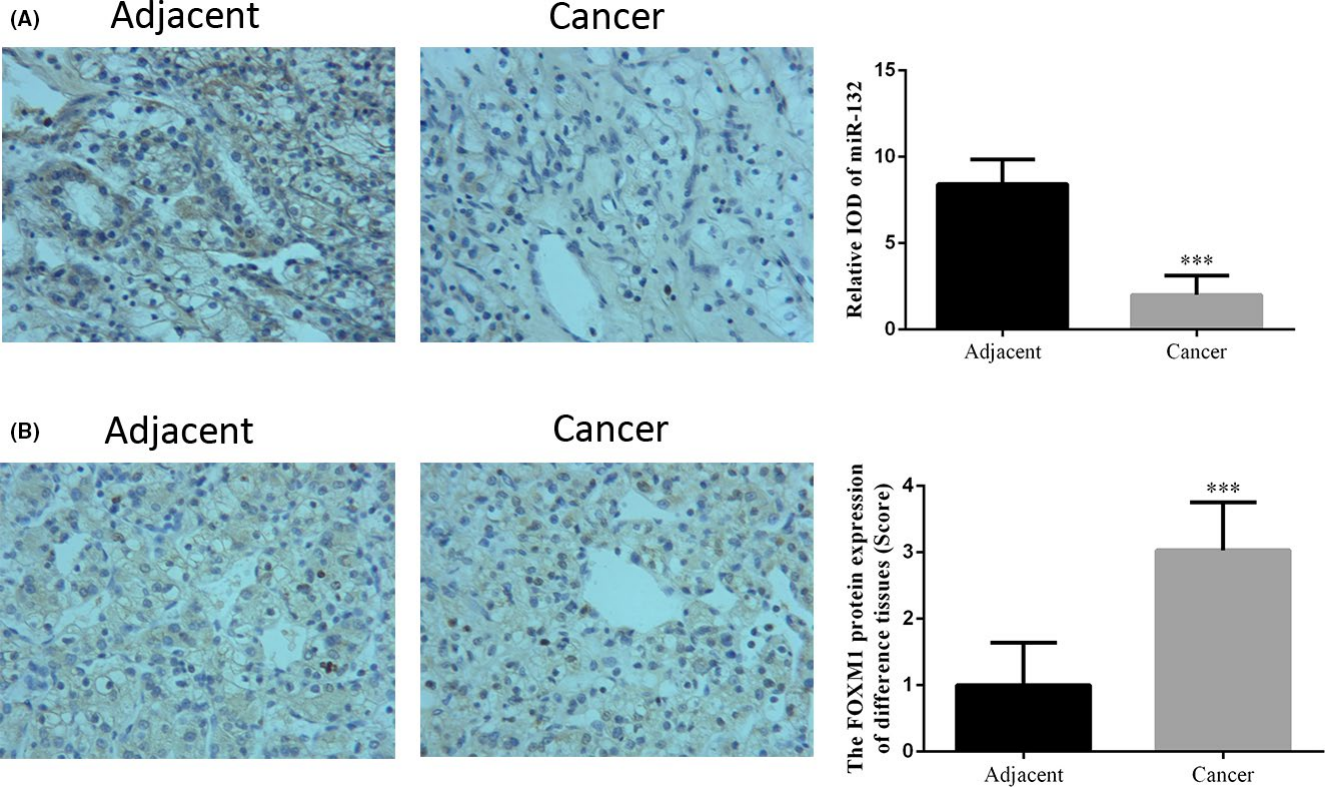
The expression levels of miRNA‐132 and FOXM1 in normal tissues and RCC tissues. The miRNA‐132 expression in normal tissues and RCC tissues was measured by ISH assay (A). The FOXM1 protein expression in normal and RCC tissues was determined by IHC assay. (B). I_OD_, integral optical density.***P < 0.05, compared with NC group

**Figure 2:**
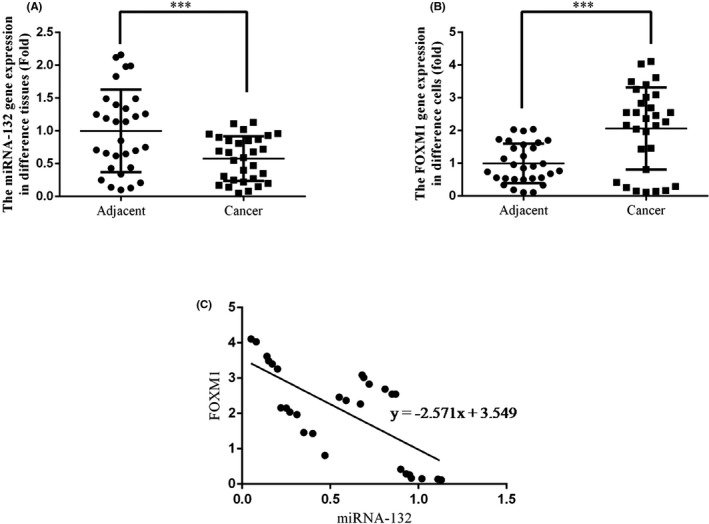
The correlation between miRNA‐132 and FOXM1 in normal tissues and RCC tissues. A, the miRNA‐132 expression of different tissues was tested by RT‐PCR. B, the FOXM1 mRNA expression of different tissues was measured by RT‐PCR ***, *P* < 0.05, compared with NC group. C, the correlation between miRNA‐132 and FOXM1 in cancer tissues was analyzed

By ISH assay, it was found that the integral optical density (IOD) of miRNA‐132 in RCC tissues was significantly down‐regulated compared with adjacent normal tissues, indicating the suppression of miRNA‐132 expression (*P* < 0.05, Figure 1A). According to the IHC assay, the results suggested that FOXM1 protein expression was significantly up‐regulated in RCC tissues compared with adjacent normal tissues (*P* < 0.05, Figure 1B). Further, RT‐PCR was used to determine the expression of miRNA‐132 and FOXM1. It was found that, compared with adjacent normal tissues, the expression of miRNA‐132 was markedly decreased (*P* < 0.05, Figure 2A) while FOXM1 mRNA expression was significantly increased in RCC tissues (*P* < 0.05, Figure 2B). By analyzing the correlation between miRNA‐132 and FOXM1, we found that the expression of miRNA‐132 was negatively correlated with the expression of FOXM1 in RCC tissues (*r *= −0.389, Figure 2C).

### 3.2 Effects of miRNA‐132 on the cell viability of KETR‐3 cells

**Figure 3:**
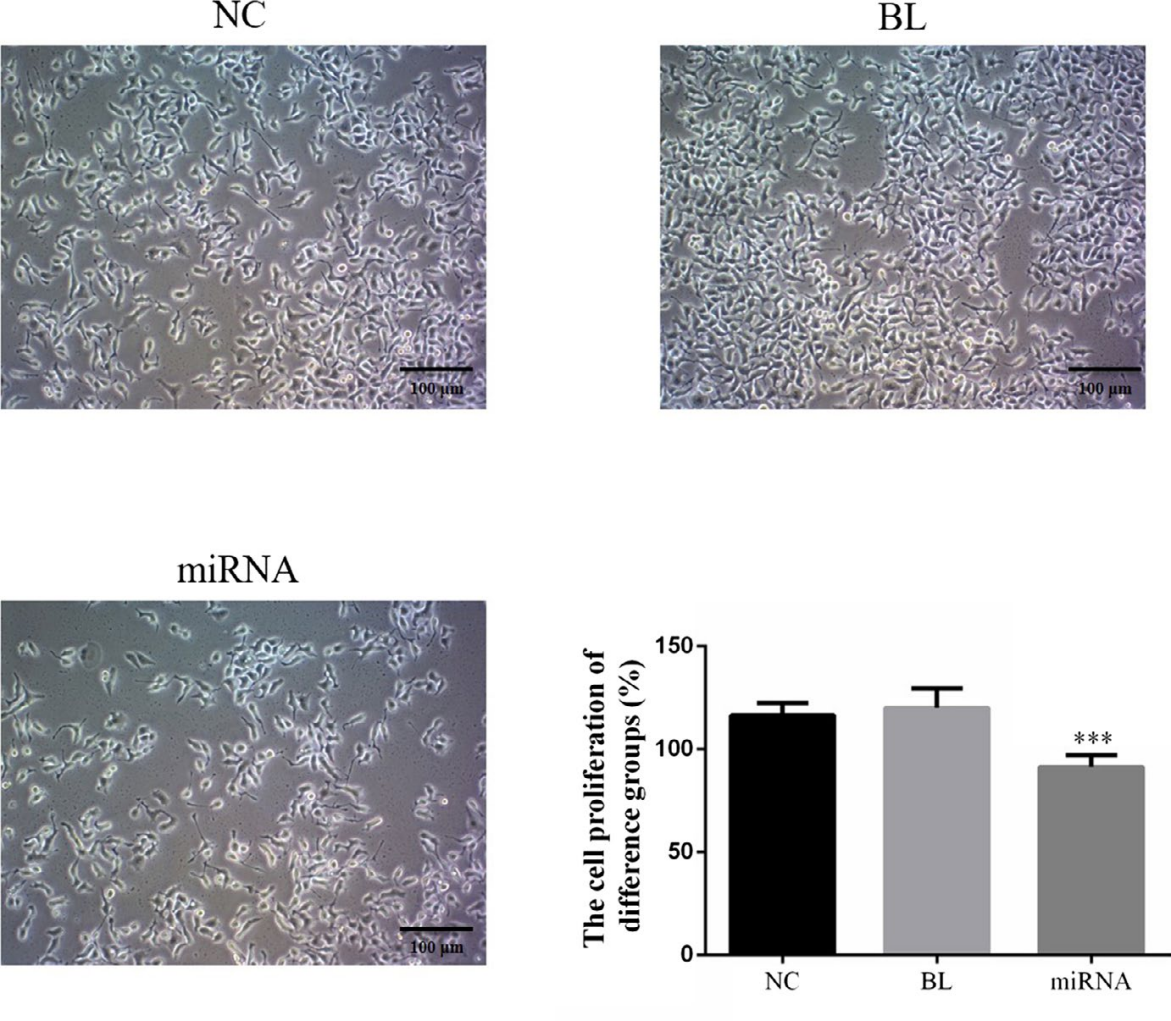
Effects of miRNA‐132 on cell viability of KETR‐3 cells. After KETR‐3 cells were transfected with miRNA‐132 by using Lip2000^TM^, cell viability in different groups was determined by MTT. ***P < 0.05, compared with NC group

As shown in Figure 3, there was no significant difference in cell viability between NC group and BL group (*P* > 0.05), indicating that empty vector had no influence on KETR‐3 cells. However, compared with NC group and BL group, the cell viability of miRNA group was significantly suppressed by miRNA‐132 (*P* < 0.05).

### 3.3 Effects of miRNA‐132 on invasion of KETR‐3 cells

**Figure 4:**
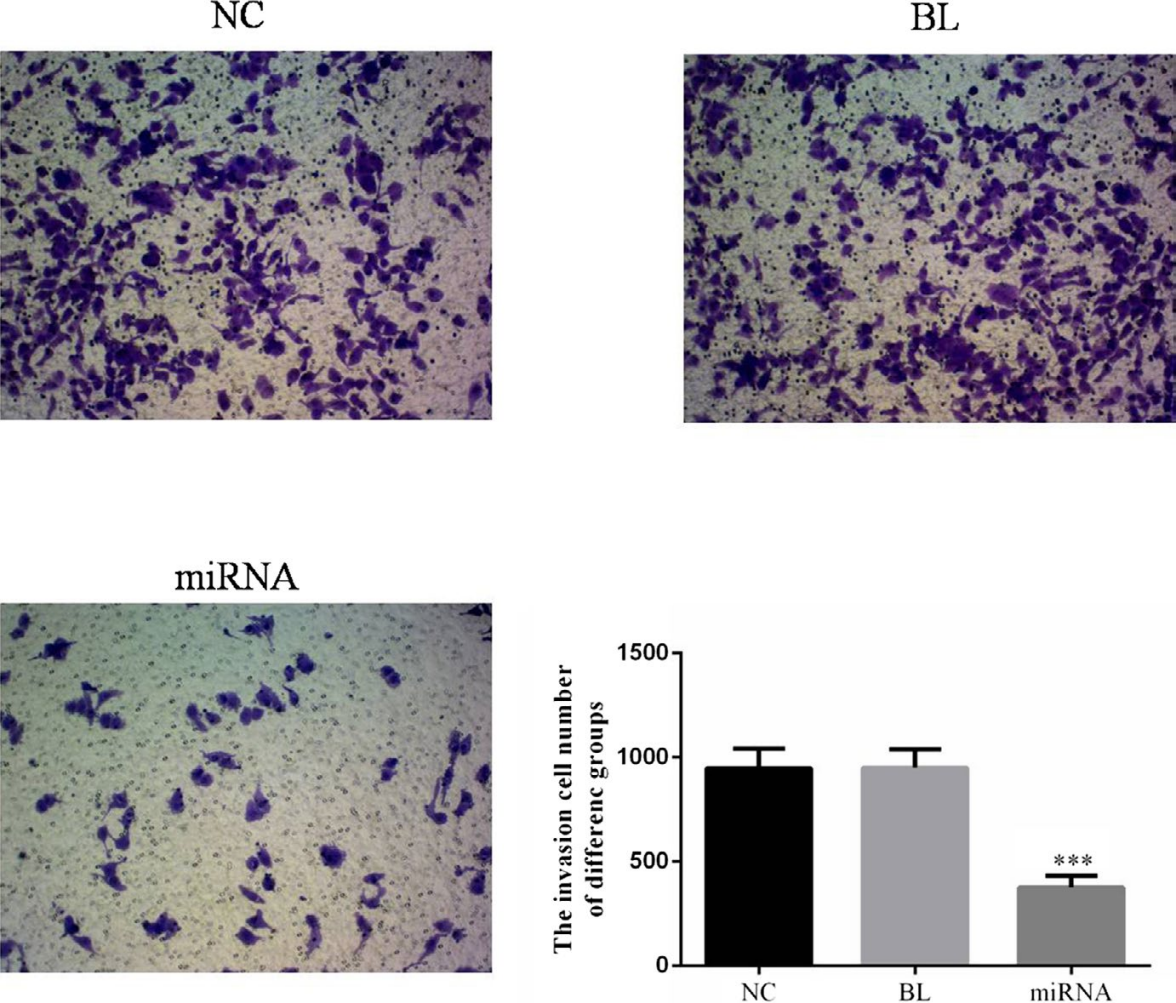
Effects of miRNA‐132 on the invasion of KETR‐3 cells. The invasion of KETR‐3 cells in different groups was measured by transwell assay following the treatment of miRNA‐132. ***P < 0.05, compared with NC group

By transwell assay, it was found that there was no significant difference in KETR‐3 cell invasion between NC and BL groups (*P* > 0.05, Figure 4). Moreover, the invasion of KETR‐3 cells in miRNA group was significantly inhibited compared with NC group (*P* < 0.05, Figure 4).

### 3.4 Effects of miRNA‐132 on the wound healing rate of KETR‐3 cells

**Figure 5:**
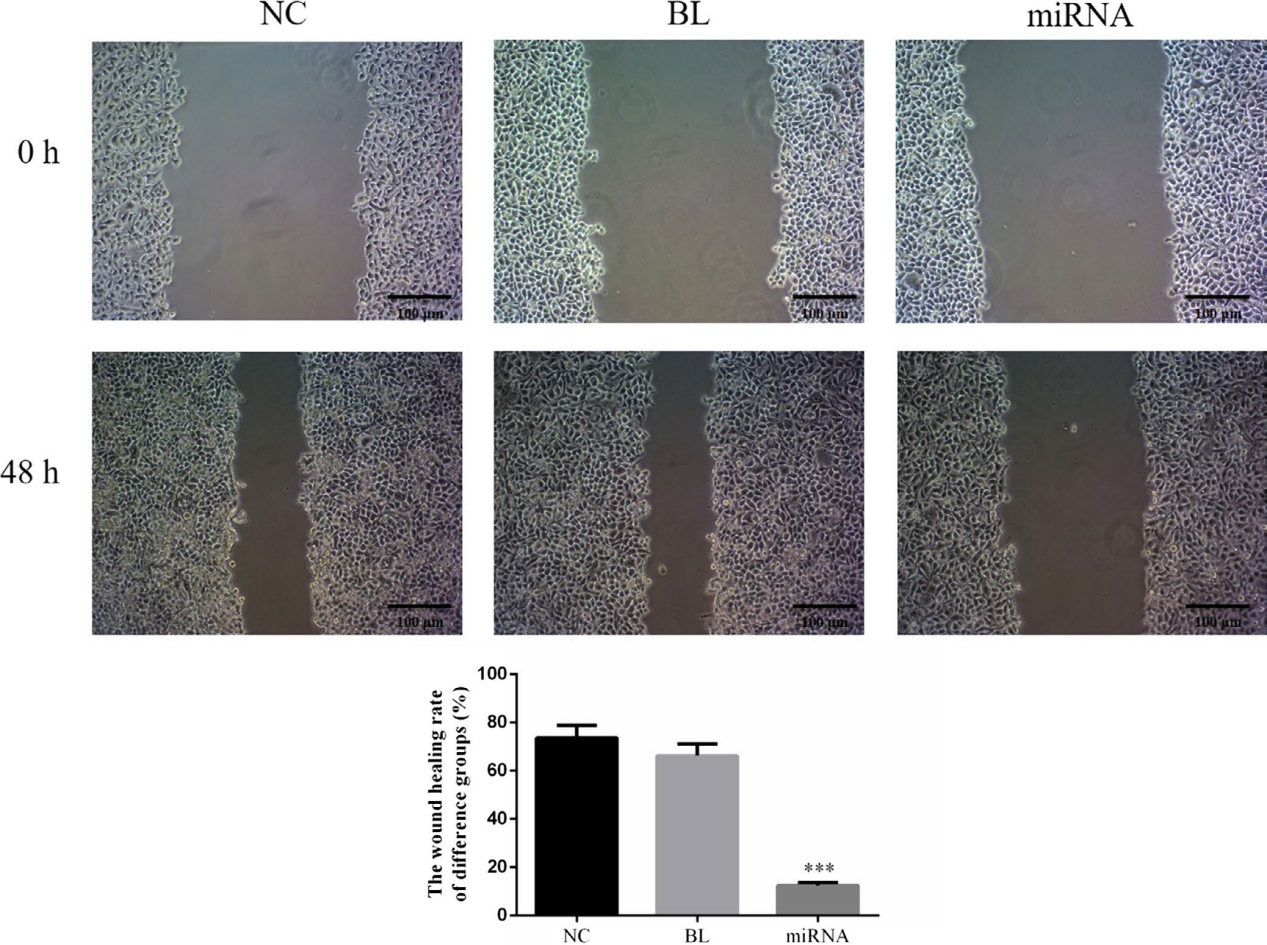
Effects of miRNA‐132 on the wound healing rate of KETR‐3 cells. By wound healing method, the wound healing rate in different groups was provided following miRNA‐132 overexpression. ***P < 0.05, compared with NC group

The effect of miRNA‐132 on wound healing rate of KETR‐3 cells was measured after scratching. Compared with NC group, the wound healing rate showed no significantly change in BL groups (*P* > 0.05, Figure 5), but that of miRNA group decreased significantly (*P* < 0.05, Figure 5).

### 3.5 Effects of miRNA‐132 on relative protein expression levels

**Figure 6:**
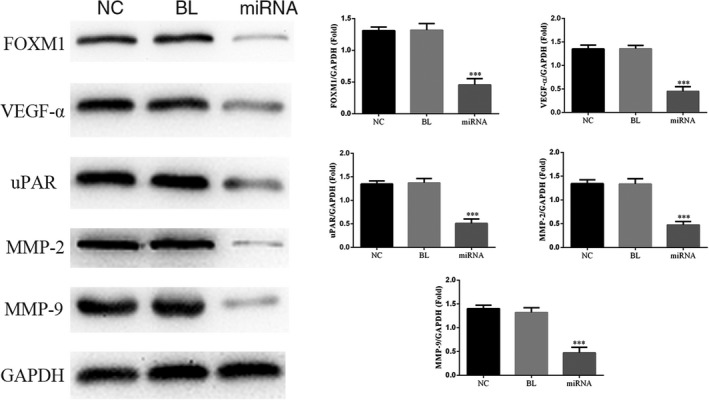
Effects of miRNA‐132 on the relative protein expression levels. After KETR‐3 cells were treated with miRNA‐132, the protein expression levels of VEGF‐α, uPAR, and MMP‐2/9 in different groups were determined by Western blot assay. ***P < 0.05, compared with NC group

The relative protein expression levels measured by Western blot assay were shown in Figure 6. Compared with NC group, the relative protein expression levels of FOXM1, VEGF‐α, uPAR, MMP‐2, and MMP‐9 in miRNA group were significantly down‐regulated (*P* < 0.05, respectively). However, there was no significant difference between NC and BL groups (*P* > 0.05).

## 4 DISCUSSION

miRNA is a class of non‐coding single‐stranded RNA molecules.^17^ miRNA binds to untranslated region (UTR) of target gene and regulates the expression of target gene at the posttranscriptional levels.^18^ It is now found that most protein‐coding genes in mammals are regulated by the expression of miRNAs. Also, the majority of mammalian protein‐coding genes are regulated by the expression of miRNA.^19^ miRNA is widely involved in various physiological activities such as cell growth, development, and apoptosis.^20^ The abnormal expression of miRNA is closely related to the occurrence of various malignant tumors, such as liver cancer, lung cancer, and breast cancer.^21^, ^22^, ^23^ The abnormal expression of miRNA is also found in RCC.^24^, ^25^, ^26^, ^27^ However, the expression of miRNA‐132 in RCC and its pathogenesis are still not clear. Therefore, studies on miRNA‐132 are expected to reveal the pathogenesis of RCC and to provide an effective method for early diagnosis and treatment of RCC.

In our present study, we find that miRNA‐132 was significantly down‐regulated in RCC tissues compared with adjacent normal tissues. Meanwhile, the expression of FOXM1 was stimulated in RCC cancer tissues with the decrease in miRNA‐132 expression. We infer that FOXM1 upregulation may closely correlated with miRNA‐132 suppression in RCC tissues. In cell experiment, the results showed that miRNA‐132 overexpression had inhibitory effects on cell viability, invasion, and migration in the RCC cell line KETR‐3 cells.

In another experiment, we explain the mechanism of miRNA‐132 in RCC. FOXM1 belongs to the transcription factor family of Forkhead, which regulates the transition phase of G1 phase of cells, and then affects cell mitosis and plays an important role in the cell cycle.^28^, ^29^ FOXM1 is mainly expressed in fetal tissues, and its expression may play a role in maintaining human tissue proliferation of.^30^ Inhibition of FOXM1 expression can lead to changes in biological behaviors such as cell growth, migration, and invasion. Studies have shown that FOXM1 is highly expressed in tumor cell lines, and tumor cells exhibit premature senility caused by anti‐apoptosis or oxidative stress and are highly resistant to chemotherapy and drug resistance.^31^ FOXM1 may be involved in the progression of human cancers.^32^ Previous studies have demonstrated that overexpression of FOXM1 inhibits the senescence of gastric cancer cells, depending on p27kip1.28.^33^ In addition, FOXM1 can mediate the formation, growth, and metastasis of tumor‐associated blood vessels in tumor tissues.^34^ Relative numerous studies have found that VEGF‐α is closely correlated with cancer invasion and migration under FOXM1 stimulation.^35^, ^36^ Meanwhile, another three important factors, uPAR, MMP‐2/9, are also closely correlated with the invasion and migration of cancer cell.^37^, ^38^, ^39^ In this study, we suggest that the protein expression levels of FOXM1, VEGF‐α, uPAR, and MMP‐2/9 were suppressed with miRNA‐132 overexpression.

In conclusion, miRNA‐132 inhibits cell viability, invasion, and migration of KETR‐3 cells and suppresses relative protein expression levels of FOXM1, VEGF‐α, uPAR, and MMP‐2/9. It suggests that miRNA‐132 might have anti‐tumor effects and can be used to treat RCC.

## CONFLICT OF INTEREST

The authors agree to share the data and materials of this study.
